# Concentrated Organic Remedies

**Published:** 1867-07

**Authors:** F. R. Payne

**Affiliations:** Marshall, Ill.


### CHICAGO MEDICAL EXAMINER.

N. S. DAVIS, M.D., EDITOR.

VOL. VIII. JULY, 1867. NO. 7.

### Original Contributions.

### ARTICLE XXVIII.

### CONCENTRATED ORGANIC REMEDIES.

BY F. R. PAYNE, M.D., Marshall, Ill.

Read before the Clark County Medical Association, April 3d, 1867.

There is no branch of human knowledge so much indebted to chemistry for practical developments as that of medicine. The chemist, with his laboratory, is able to present to the profession the active principles of plants, either combined or separate. It is, certainly, very desirable for the practitioner to possess the active principles of the vegetable kingdom in a concentrated form. It is equally important that we secure these remedies in a durable form, and of uniform strength, containing all the medicinal principles of the plant. By this means, the stomach receives nothing but that which is necessary for the removal of disease. It has been demonstrated, by chemical analysis, that vegetables contain resinoids, neutrals, oils, and alkaloids, and in these elements reside the therapeutic properties. It is the object of this paper to briefly speak of a few of the comparatively new remedies which have been prepared in powdered form, and represented as containing all the medicinal principles of the roots or plants from which they are obtained.

The first to which we will refer, is the

GELSEMIN.

This a remedy derived from the bark of the root of the common woodbine, or yellow jessamine, and contains three principles, resinoid, neutral, and alkaloid. Its properties are febrifuge, nervine, anti-spasmodic, relaxant, and some claim that it is alterative and ecbolic. If the remedy possesses all of these properties, it may, with every promise of success, be employed in the treatment of all febrile diseases, pneumonia, pleuritis, acute rheumatism, gonorrhœa, chorea, epilepsy, convulsions, in short, all inflammatory and nervous diseases. I cannot, by my limited experience in its use, testify to its value in this wide range of human maladies, but feel sure that it is one of the best arterial sedatives known to the profession. It is a positive and active therapeutic agent, and by isolating the three principles of the plant they are recombined and form a beautiful and durable powder, with a definite and uniform standard of strength. The average dose of gelsemin is one-half grain, but in some cases, owing to constitutional peculiarities, it may require two grains. If it is not given in sufficiently large doses to produce its constitutional effects, it will not fully accomplish the object for which it was administered. It must be given in doses that will cause slight dimness of vision and double-sightedness, but in no case should the remedy be carried to complete prostration of the muscular system. Physicians who have used this remedy extensively state, that even when carried to complete exhaustion, no permanently injurious effects are produced. The beneficial effects of this remedy are often manifested before constitutional symptoms are produced. When the dose is large enough to reduce the pulse to its normal standard it is not prudent to increase; but the remedy ought to be continued for some time, in small doses, after the action of the heart is controlled. I have used this remedial agent in pneumonia, typhoid fever, convulsions in children, produced by excessive febrile excitement, and, in fact, in all cases of fever where the pulse exceeds 100 per minute, and can testify to its value in these diseases. It is, undoubtedly, a valuable remedy, and it is in a convenient form, which is a very important consideration to the country physician, who is not only called upon to prescribe, but to dispense the medicines recommended. It is not a specific, but a positive and powerful agent, consequently not to be trifled with or incautiously used. When given at the proper time and in small doses, it is as harmless as any other potent drug in the materia medica.

CAULAPHYLLIN.

This is the blue cohosh, or squaw root. It has two principles, according to the analysis of B. Keith, consisting of a resinoid and neutral. It is claimed by some who use this remedy, that it is a valuable antispasmodic, alterative, tonic, ecbolic, diaphoretic, diuretic, and vermifuge. The average dose is three grains. It is said that this dose may be repeated every hour or two with perfect safety, and even increased to five or ten grains. This remedy is highly extolled by G. COE, M.D., and others, but we cannot testify to its value. We have used this preparation, prepared by Dr. F. D. HILL, Cincinnati, and feel confident that it is not entitled to the reputation claimed by this school of exclusive remedies. It is only a temporary remedial agent. If this preparation is a fair representative of the medicine, it is not entitled to the favorable consideration of the profession. We are willing to use all therapeutic agents which, by experience and proper test, proves to be valuable. We have used the remedy in combination with others, but do not regard it as even a good auxiliary remedial agent.

LUPULIN.

This is derived from the common hop. The part used is the strobiles, or cones, and is said to contain three principles, resin, resinoid, and neutral. Its properties are nervine, febrifuge, hypnotic, diuretic, and tonic. When it is employed, you may, with confidence, expect beneficial results. When given to small children, we have noticed that it frequently promotes diaphoresis and diuresis, and often succeeds in the production of refreshing sleep. It is also a valuable tonic, mild in its operation, and valuable in many forms of indigestion, and especially where there is a tendency to gastritis. It allays and soothes the irritability of the mucous tissues, and thus aids in the process of digestion. I have used this remedy, and feel confident that it is a valuable auxiliary remedial agent. The lupulin usually found in commerce, is nothing more than the pollen of the flowers. The best preparation contains all the medicinal virtues of the hop, which reside not only in the pollen, but also in the parenchyma.

The dose of *true* lupulin is from two to five grains. We have used it in febrile diseases in children, and if we have a genuiue preparation, it will in many cases produce hypnotic and febrifuge effects. It is especially valuable in diseases of children, where we desire an anodyne and cerebral excitement precludes the use of opium and its preparations. It also possesses other advantages as a substitute for opium, by not disturbing the stomach, or producing constipation. We have noticed that when this remedy fails to produce hypnotic or anodyne effects, it proves a valuable diuretic; I have frequently had patients say that it did not make them sleep, but caused a free flow of urine. The celebrity and value of common *hop-beer*, as a tonic, nervine, and diuretic, depends upon the lupulin it contains. The good or bad quality of the beer depends upon the quality and quantity of lupulin used in its preparation.

We have used lupulin with good results in nervous headache, hysteria, chronic cough, and suppression of urine. It is highly recommended as a valuable remedy in spermatorrhœa, but we have not tested its virtues in this disease. It is certainly a good remedy for irritability of the mucous tissues. It is very important to get a genuine article, and not simply the pollen of the flowers.

SANTONIN.

This is a remedy obtained from the *artemisia santonica*, or mugmort. It is a peculiar, white, crystalline substance, soluble in ether or alcohol, and almost tasteless. It is a native of Europe, but is now being cultivated in the United States. We also have the brown, or impure santonin, which is derived from the Aleppo wormseed. This is cheaper than the former. The properties of this valuable concentrated remedy are said to be tonic and narcotic, but its chief value consists in its anthelmintic virtues. I have used it extensively for the last six years, and feel warranted in stating that it is the best remedy known for the destruction of the acaris lumbricoides. It is an active and powerful remedial agent, but when properly administered it is not only perfectly safe but reliable.

It may be administered alone, or in combinations with other agents. When children are troubled with worms, the symptoms of any disease with which they are attacked are aggravated by the presence of these intestinal parasites, and we can use the santonin in combination with the appropriate remedies for the disease. Our favorite mode of prescribing this medicine is as follows, for a child say three years old:—

℞. Santonin,------------------------------ grs. iij.
Hyd. cum creta,---------------------- " vi.
Dover Powd,-------------------------- " ij.

Mix, and make three powders. Give one every three hours, and four hours after last work off with *oil castor* and a few drops turpentine.

This prescription will never fail to expel lumbricoides if they are in the intestines. We have frequently administered one or two doses of santonin alone, and often it has been followed by the expulsion of from six to twenty worms, and this result was accomplished without the subsequent use of a cathartic. For several years, we have almost invariably added santonin to the first cathartic powders given to children, in the treatment of any disease with which they have been attacked, and, oftentimes, where the existence of worms is not anticipated, large numbers will be expelled. We can, with perfect confidence, recommend santonin as one of the most convenient and reliable anthelmintics belonging to that list of remedies.

When properly administered, no injurious effects can possibly result from its use. If given in large doses, it will produce some cerebral excitement, but, according to our observations, its specific action is fully obtained without the production of its constitutional effects. We earnestly recommend this remedy to all practitioners who have not yet used it.

ASCLEPIN.

This is the active principles of the *asclepias tuberosa*, or pleurisy root. The part used is the root, and it contains two principles, resinoid and neutral. Its properties are said to be alterative, antispasmodic, carminative, diaphoretic, diuretic, expectorant, laxative, and tonic. If it possessed all these properties, it might, with every hope of success, be employed in all fevers, pneumonia, pleuritis, rheumatism, colic, colds, and inflammatory diseases, of whatever type.

We have used this remedy in many cases, and feel sure that it is of but little, if any, value. We have never been able to notice any good effects result from the use of the preparation prepared by Dr. Hill. During the last six years, we have given it a fair and impartial trial, but have invariably been disappointed. If the article we used is a fair representative of the remedy, it is not even entitled to a place in the materia medica.

PHYTOLACCIN.

This is obtained from the root of the *phytolacca decandria*, or common poke root. It has two principles, resinoid and neutral, and its properties are alterative, deobstruent, and diuretic; in large doses, emetic and cathartic. It may, with every prospect of success, be employed in the treatment of rheumatism, glandular, and cutaneous diseases.

We have found it a potent and reliable agent in the treatment of diseases where there is tardiness of action on the part of the secreting, absorbing, exhaling and eliminating vessels.

It is a powerful remedy, and ought to be administered with great care. From one to two grains, three times a day, it proves a good, safe, and efficient resolvent, and manifests its influence in all the glands of the body. In the chronic form of rheumatism, it is a remedy of decided utility. We have used the following combination in cases of chronic rheumatism, with good effects:—

℞. Phytolaccin, -------------------------

Podophillin, ------------------------ āā ℈j.

Gelsemin, ------------------------- grs. x.

Mix, and make twenty powders, and give one every six hours. If this nauseates the stomach or acts too vigorously on the bowels, we diminish the dose of the two first and add $\frac{1}{8}$ grain morphine to each powder.

We have never thoroughly tested the virtues of this remedy in any other disease, but feel confident that it might with propriety be used in enlargement of the spleen and hepatic torpor. It is highly recommended by some, as a valuable remedy in tuberculosis, gonorrhœa, leucorrhœa, and carcinomatous affections, but in the latter it is used externally.

In using these concentrated remedies, it is vastly important for the physician to procure genuine articles. Some chemists prepare them in such form that the doses above indicated are too large; at the same time, other preparations are not of sufficient strength. It is, therefore, left to the judgement of the practitioner to decide the propriety of quantity and repitition of the article used. It seems to be a fact, fully established, that compound vegetable substances which contain only a small number of elements and atoms are more durable than those more complicated. In proportion to the multitude of the atoms and principles that enter into the composition of organic compounds, we have an increased disposition to undergo transformation and decomposition. This fact is important to those who desire to procure the active medicinal principles of plants. It is hoped that we will soon be presented with the active principles of the following crude remedy:—

POLYTRICHUM JUNIPERUM.

This is the common *hair-cap moss.* It is an evergreen and indigenous plant, and there can be no doubt, with those who test its virtues, that it is a valuable diuretic. It grows on high, dry places, along the margin of woods and exposed places, and seems to prefer a poor, sandy soil.

We have used it with decided benefit in many dropsical cases.

The dose is about two fluid ounces of the effusion every half-hour. It is very highly recommended by some medical men, and we feel confident that it is a better, or at least more active diuretic than uva ursi or buchu leaves. Some physicians assert that they have, with this remedy, removed from dropsical patients, in twenty-four hours, forty pounds of water.

From what we know, by experience in the use of this agent, we can with confidence recommend it to the profession as a pleasant, safe, and reliable diuretic. It is hoped that, by the aid of chemical laboratories, we will soon be presented with the active principles of this plant in a concentrated form.

Every country practitioner can readily realize the disadvantages of crude medicines. In this land of mud, it is very inconvenient to carry huge pill-bags, pregnant with crude roots and herbs; but this is a small matter, when compared to the inconvenience of patients swallowing pints and quarts of decoctions and infusions, in order to get the proper dose of a remedy.

